# Progress of Adipokines in the Female Reproductive System: A Focus on Polycystic Ovary Syndrome

**DOI:** 10.3389/fendo.2022.881684

**Published:** 2022-05-26

**Authors:** Peipei Chen, Rui Jia, Yuanyuan Liu, Mingya Cao, Liang Zhou, Zhiming Zhao

**Affiliations:** Department of Reproductive Medicine, The Second Hospital of Hebei Medical University, Shijiazhuang, China

**Keywords:** PCOS, adipokines, female reproductive system, reproductive endocrinology, insulin resistance

## Abstract

Adipose tissue, one type of loose connective tissue in the human body, maintains the primary task of energy storage. Adipose tissue is not only an energy reservoir but also plays a vital role as the largest endocrine organ of the whole body *via* releasing a variety of adipokines, which participate in many pathophysiological processes, such as energy metabolism regulation, glucose and lipid metabolism, and inflammation. Polycystic ovary syndrome (PCOS) is a disorder that mainly involves the female reproductive system, affecting women of childbearing age particularly. Insulin resistance (IR) and hyperandrogenemia (HA) have been implicated as a critical link involving the etiology and outcome of PCOS. A great deal of studies has bridged the gap between adipokines (such as Adiponectin, Chemerin, Metrnl, Apelin, Resistin, Visfatin, Leptin, Vaspin, Lipocalin 2, and Omentin) and reproductive fitness. In this review, we will focus on the adipokines’ functions on PCOS and come up with some points of view on the basis of current research.

## Introduction

Polycystic ovary syndrome (PCOS) is characterized by heterogeneity, estimated to jeopardize 5.6% (1) nationwide and 6% - 20% (2) worldwide child-bearing women with the tendency to become an epidemic. The most commonly used criteria for PCOS come from the Rotterdam ESHRE/ASRM-Sponsored PCOS Consensus Workshop Group (3): oligo- or anovulation, clinical and/or biochemical signs of hyperandrogenism, and polycystic ovaries after exclusion of other etiologies (congenital adrenal hyperplasia, androgen-secreting tumors, or Cushing’s syndrome). There has been a gradually increasing awareness that PCOS is likely to be a complicated polygenic disease, affected by external factors like lifestyle and dietary habit. Clinical manifestations of PCOS center on endocrine metabolism and involve reproduction, endocrine, substance metabolism, and psychological health although with an unclear etiology. Endocrine changes in PCOS inflict on other female systems rather than reproduction alone. With further study on adipokines, a variety of bioactive compounds released by adipose tissue have been revealed, and the signal crosstalk between adipokines and PCOS has been gradually understood. Through the paracrine and endocrine pathways, adipokines act widely on different parts of the human body, regulating and controlling glucose and fatty acid metabolism, energy expenditure, inflammatory response, cardiovascular function, reproduction, and other biological processes over the entire body or locally (4, 5). A meta-analysis published in *Lancet* in 2021 consisting of 71 studies (including 2495 patients with PCOS and 2520 controls) revealed that non-obese PCOS patients have significantly higher circulating levels of Chemerin, Leptin, Resistin, and Visfatin but a significantly lower circulating level of Adiponectin when compared with non-obese healthy controls (6). A vast and expanding universe of research into the crosstalk of adipokines and PCOS has been issued, emphasizing the role of adipokines in the development of PCOS: metabolic disturbance like hyperinsulinemia, IR, HA, ovary dysfunction like granulosa cell apoptosis, and abnormal endometrial receptivity accompanied with adverse pregnancy outcomes during assisted reproductive technology (ART).

## Methods

All documents were obtained from a systematic search of articles reported in the PubMed. The key words used were: “PCOS”, “adipokines”, “Adiponectin”, “Chemerin”, “Metrnl”, “Apelin”, “Resistin”, “Visfatin”, “Leptin”, “Vaspin”, “Lipocalin 2”, “Omentin”, “genetic variants”, and “polymorphisms”. Original articles, meta-analyses, and review articles in English were included in this review. Topics about adipokines, their genetic polymorphisms, biological function, and role in the physiopathology of PCOS and their relationship with the outcomes of ART of PCOS were addressed in this review.

### Adiponectin

As a collagen-like 30-kDa protein, Adiponectin (also known as acrp30, apM1, adipo-Q, or GBP28) is the most abundantly secreted adipokine by the adipose tissue, mainly the white adipose tissue, to travel through the blood (7). It is clearly a distinct member of a protein family characterized by a collagenous helical structure at the NH2 terminus and a globular domain at the COOH terminus (8). The Adiponectin gene in humans is located in the long arm of chromosome 3 (3q27), containing three exons and two introns (9). The study by Yamauchi and colleagues (10), which has discovered Adiponectin receptors 1 and 2 (AdipoR1 and AdipoR2), was a Big Bang. From then on, a vast and expanding universe of research on Adiponectin has been issued. Secreted by adipocyte as a metabolic messenger, Adiponectin targets human organs and tissues including the reproductive system by binding to its receptors (11) and participating in various physiological and pathological processes as an initiating factor or intermediary. Adiponectin is involved in the pathophysiological processes of many diseases, inhibiting tumor cell growth and metastasis through anti-proliferation and induction of apoptosis (12, 13). It has been used in the prevention and treatment of type 2 diabetes mellitus (T2DM) and atherosclerosis *via* anti-inflammation and improvement of insulin sensitivity (14, 15) as well as arthritis (16).

Adiponectin and its receptors (AdipoR1 and AdipoR2) have been found in human ovarian cells (oocytes, granulosa cells, follicular membrane cells, and cumulus cells) and human follicular fluid at various stages of follicular development (17), combining with each other to regulate follicular development and ovulation through increasing the gonadotropin (Gn) or insulin sensitivity directly or indirectly (18). For one thing, Adiponectin can relive the ovulatory event of porcine *in vitro* by directly acting on the porcine granulosa cells, accompanied by continuous and rapid induction of cyclooxygenase-2 (COX-2) and its downstream substances along with the activation of synthase (PGE synthase) in cytoplasm (19). For another, Adiponectin decreased the production of progesterone and androstenedione in insulin-induced bovine follicular theca cells (TCs) through inhibition of cytochrome P450 17A1 (CYP17A1) and cytochrome P45011A1 (CYP11A1) (20), leading to reduced steroidogenesis. As is known to all, the mid-menstrual (LH) peak induces the activation of a series of signaling pathways associated with ovulation, leading to rupture of the follicle and release of the oocyte. Given all this, Adiponectin plays a significant role in ovulation by regulating the steroid production of granulosa cells (GCs) and follicular TCs, expansion of cumulus cells, and an interaction with luteinizing hormone (LH) even though the specific mechanisms need further investigation.

In addition to being expressed in the ovaries, Adiponectin has also been found to be expressed in the hypothalamus and pituitary gland. Short-term treatment with recombinant Adiponectin can inhibit gonadotropin-releasing hormone (GnRH) secretion by activating AMP-activated protein kinase (AMPK), thereby reducing LH secretion (21). Correspondingly, Adiponectin production can be induced in human ovaries after treatment with recombinant LH (22). A hypothesis is thus suggested that there may be a regulatory mechanism of Adiponectin, similar to that of the sex hormone axis *in vivo*: GnRH and Gn induce local Adiponectin production in ovaries while circulating high levels of Adiponectin in turn inhibit GnRH and LH production in the hypothalamus and pituitary gland through negative feedback.

The latest meta-analysis shows that there is a significantly lower circulating Adiponectin level in non-obese PCOS patients (6), and the serum Adiponectin level is negatively correlated with the insulin and IR index after adjusting for body mass index (BMI) confounders (23). In addition, Adiponectin levels were lower in the non-ovulating PCOS patients than in the regular ovulating PCOS patients (24), indicating its important role in ovulation disorders of PCOS. Combined with previous animal studies which revealed Adiponectin inhibited LH and androgen secretion but promoted ovulation, it is speculated that the low Adiponectin level in PCOS may negatively increase LH secretion in the pituitary gland, resulting in a high LH and androgen level and ovulation dysfunction in PCOS patients. Abnormal hormone exposure in early pregnancy can affect metabolic status in adulthood. Most women with PCOS have unexplained high androgen levels during pregnancy (25, 26), and their offspring will exhibit HA and metabolic disorders which are centered on IR in adulthood. Animal studies have shown that embryonic Adiponectin supplementation improves the metabolic syndrome in adult PCOS offspring by activating the PI3K-Akt pathway (27). In PCOS patients treated with metformin, rosiglitazone, and pioglitazone, the serum Adiponectin levels increased, accompanied by decreased insulin secretion, enhanced insulin action, and increased lipid oxidation (28), suggesting that Adiponectin is a beneficial factor for human body and is involved in regulating the function of the female reproductive system.

Although obese PCOS patients have a certain natural pregnancy rate after losing weight, most PCOS patients with infertility need assisted reproduction. Unfortunately, there is relatively little research in this area. Inal et al. (29) found that although PCOS patients had a low serum Adiponectin level, there was no significant difference in the embryo development quality or clinical pregnancy rate between the PCOS and non-PCOS patients during ART. This may be related to the improvement of HA and IR that benefits from physical exercise and improved diet during pregnancy medication. Although a certain relationship between the serum Adiponectin level and pregnancy outcome in PCOS patients has not been revealed, the close relationship between the Adiponectin level and the characteristic pathological changes of PCOS has been confirmed. Therefore, the specific mechanism of Adiponectin in the development of PCOS is worth studying because it has a broad prospect.

Regarding the effect of Adiponectin gene polymorphisms on PCOS, current studies showed a close correlation with race. The rs1501299 single nucleotide polymorphism (SNP) of Adiponectin was significantly correlated with PCOS risk in East Asians (30, 31), the rs17300539 showed a correlation with PCOS risk in a Chinese population (32), and rs1501299 and rs2241766 polymorphisms both had a close correlation with PCOS risk in Caucasians (33). In addition, several studies have shown the association of genetic polymorphisms of Adiponectin with endocrine metabolism and steroid hormone production in PCOS patients (34, 35). All these studies demonstrated a relationship between PCOS and Adiponectin at the genetic level.

### Chemerin

Chemerin was first discovered in 1997 while studying psoriasis and was named tazarotin-induced gene 2 (TIG2) because it could be induced by TIG2 under certain conditions (36). In 2003, the ligand was identified as the orphan receptor ChemR23 and formally named Chemerin, which turned out to be an adipokine (37–39). Located on human chromosome 7, the Chemerin gene consists of five coding exons. It is transcribed primarily by the white adipose tissue and binds to three receptors: chemokine-like receptor 1 (CMKLR1), G protein-coupled receptor 1(GPR1), and CCRL2 (e.g., 2 CC motif chemokine receptor), though binding G proteins to perform their physiological functions (40–44). Chemerin has been found to play a key role in metabolic diseases, such as obesity and T2DM. It also participates in diseases of multiple systems, such as autoimmune diseases (45), urinary diseases (46), digestive diseases (47), and psychiatric diseases (48), by regulating blood pressure, inflammation, immune response, fat cell differentiation, and carbohydrate metabolism.

Chemerin is expressed in basal cells of the human uterus, stromal cells, and extravillus trophoblast cells of pregnant women and the human placenta (49–51). Furthermore, Chemerin and CMKLR1exist and play an active role in human GCs (42). It is an indisputable fact that local IR exists in GCs of human ovary from PCOS patients. Excessive insulin in serum can promote Chemerin production (52), and PCOS patients have elevated levels of Chemerin which are associated with IR, as compared with non-PCOS patients. Follicular fluid and luteinized granulosa cells of PCOS patients with IR have higher Chemerin levels, which can be induced by over-expressed insulin and in turn damage the glucose uptake capacity through affecting Insulin Receptor Substrate 1/*2* Irs1/2, Akt phosphorylation, and GLUT4 translocation, finally aggravating IR (53). This is a vicious cycle between Chemerin and IR. A low progesterone level observed in PCOS serum indicates unexpected ART outcomes. Estienne et al. demonstrated that the over-expression of Chemerin system in human luteinized granulosa cells (hlGCs) in the case of PCOS could be a reason for progesterone secretion disturbances through CMKLR1, STAR, MAPK3/1, and PRKAA (54). They observed a significantly higher concentration of Chemerin and CMKLR1 within the follicular fluid and hlGCs at both the gene and protein levels from PCOS patients. Furthermore, incubation with the CA4910 nanobody, a biological fragment raised against CMKLR1, abolished the inhibition of Chemerin-induced STAR mRNA expression as well as progesterone secretion in human granulosa cell line KGN and primary hlGCs from PCOS patients. However, no changes were found about the CYP17A1, a key enzyme that converts cholesterol into progesterone, suggesting that Chemerin may lead to a low progesterone level in PCOS patients by inhibiting cholesterol production. Chemerin has been shown *in vitro* to reduce IGF-1-induced estrogen and progesterone production and cell proliferation by binding with CMKLR1, thus reducing the activation of the IGF-1R signaling pathway in primary cultured human granulosa cells (42).

As the most abundant immune cells in the ovary, macrophages play a crucial role in the inflammatory state and clearance of senescence and apoptotic cells. HA in PCOS patients leads to increased Chemerin levels in the ovaries. In rats treated with dihydrotestosterone (DHT) for 15 days, researchers demonstrated that high levels of Chemerin as a ligand for CMKLR1-expressing monocytes in the blood resulted in local ovarian inflammation, leading to granulosa cell apoptosis, follicular growth arrest, and anovulatory infertility (55). In addition, a retrospective study confirmed the association between Chemerin and ovarian polycystic changes in PCOS patients, and the results showed that serum Chemerin concentration could reflect the severity of ovarian polycystic changes (56). All these studies taken together suggest that Chemerin plays a role in follicular dysplasia in PCOS patients, but the specific mechanism behind this phenomenon needs to be further studied. The serum Chemerin level of PCOS women with HA was higher than that of women with increased androgen level alone, consistent with the change in level of free androgen index and total testosterone level. Prenatal androgen overload can cause early life changes that affect the reproductive axis and metabolic state, contributing to the development of PCOS. The female offspring of prenatal androgen hyperactive (PH) rat models exhibit two phenotypes: irregular ovulation phenotype (PHiov) and anovulation phenotype (PHanov). Both phenotypes have lower serum Chemerin protein levels (57), which suggests that excessive androgen exposure during pregnancy may be involved in the development of PCOS by affecting the Chemerin level of female offspring.

By studying the effect of Chemerin levels on intracytoplasmic sperm injection (ICSI) results of lean patients with PCOS, Kabil Kucur et al. found that compared with the control group, the serum and follicular fluid Chemerin levels of PCOS patients were significantly higher. Among the PCOS patients under ART, patients with the successful ART had lower Chemerin levels in the serum and follicular fluid than those who failed the ART (58), indicating that Chemerin is not only involved in the pathogenesis of PCOS, but also a risk factor for the failure of the ART cycle.

Although affected by obesity status, Chemerin rs17173608 polymorphism had been shown to have a certain relationship with PCOS among Iranian women (59).

### Metrnl

Originally reported as a homologous transcription of Meteorin with a role in the central nervous system as a neurotrophic factor (60, 61), Metrnl was identified in 2014 as a new adipocytokine in a caloric restriction model. Metrnl gene was located on human chromosome 17q25.3, encoding a secreted protein containing 266 amino acids (62). Different from the high expression of Meteorin in the nervous system (63), Metrnl is more likely to be expressed in the white adipose tissue and other tissues like heart and skeletal muscle (62, 64). Current studies have shown that Metrnl can affect the nervous system, digestive system, and cardiovascular system by inducing the browning of white fat and muscle regeneration as well as antagonizing IR and anti-inflammatory as a neurotrophic factor, muscle factor, and adipokine (61, 65, 66).

Two case-control studies revealed lower serum Metrnl levels in PCOS patients than in normal controls, contrary to the level change of IR markers, FSH, and an independent correlation between Metrnl and PCOS (67, 68). A study from Iran found that serum levels of Metrnl in the infertile PCOS (PCOS-inf) and PCOS with recurrent pregnancy loss (PCOS-RPL) subgroups were significantly lower than in the control group, which remained significant after adjustment for confounding factors but disappeared when compared between the PCOS subgroups. Interestingly, after stratification by BMI, the significant difference in the serum Metrnl levels between overweight and normal-weight individuals in the PCOS-inf and PCOS-RPL groups disappeared. There was a significant rising of Metrnl levels in serum at 24 and 28 weeks of gestation in pregnant women with gestational diabetes compared with normal pregnancies, and similar results were observed in maternal peripheral blood and umbilical cord blood collected at late pregnancy. After delivery, the significant increase observed at 24-28 weeks of gestation did not exist (69), this covarying performance suggesting that Metrnl may be involved in the occurrence of gestational diabetes.

### Leptin

Leptin, encoded by a gene on the human chromosome 7 mainly coming from the white adipose tissue, was discovered as an adipokine in 1994 (70, 71). Leptin receptor, a member of the class I cytokine receptor family, is encoded by diabetes (db) gene (72). Leptin is secreted mostly by the adipose tissue into the blood and binds mainly the Leptin receptor to activate Janus kinase signal transduction transcriptional activator signal (JAK-STAT) pathway to regulate food intake and energy homeostasis (73), participating in physiological activities such as immune response, neuroendocrine response, systemic inflammatory response, and reproductive function.

Leptin can regulate reproductive function at the central level. Studies have shown that Leptin regulates GnRH secretion through neurons expressing Kisspeptin and NO, thus playing a role in the initiation of puberty and periodic secretion of Gn even though there is no Leptin receptor in GnRH neurons (74). Kucera and colleagues (75) found that increased Leptin in follicular fluid is a sensitive marker of anovulatory fertility disorders by comparing the levels of Leptin in follicular fluid in fertility disorders with different etiologies. One of the major problems confusing PCOS patients of reproductive age is anovulatory infertility. A meta-analysis showed that the circulating Leptin level in non-obese PCOS patients was significantly higher than that in obese PCOS patients (6). Besides, the relevance between homeostatic model assessment-insulin resistance (HOMA-IR) and serum Leptin level has also been elucidated (76). Insulin has been shown to enhance Leptin gene expression and elevate circulating Leptin levels (77). IR in PCOS patients leads to increased insulin content, which may induce the white adipose tissue to secrete more Leptin to participate in the development of PCOS. Studies have shown that Leptin plays a role in the occurrence and development of PCOS by regulating the reproductive endocrine axis and local steroid production of ovary as well as participating in IR. Circulating Leptin level is positively correlated with increased IFN-α, followed by apoptosis of human granulosa cells (KGN), which has been demonstrated in cell experiments (78). Obesity is another problem faced by PCOS patients, Liu et al. (79) have found that hyperandrogenism may increase feeding and lead to obesity by suppressing Leptin levels in the cerebrospinal fluid thus inhibiting Leptin signaling in the hypothalamus in a rat model.

Studies have shown that Leptin enhanced the expression of aromatase genes by activating classic signal transduction pathways MAPK and PI3K, whereas this phenomenon was significantly attenuated in granulosa cells of PCOS patients, together with significantly increased plasma Leptin and decreased soluble Leptin receptor (s OB-R) in PCOS patients (80). This may suggest that Leptin resistance in PCOS patients may be possibly mediated by Sam68, an RNA-binding protein that is widely expressed in the body and has signaling functions (81). A study aiming to illustrate the effects of aerobic exercise on PCOS rats with HA showed the effect of Leptin on inflammation in PCOS (82). In this study, the researchers divided the study subjects into normal control group with or without aerobic exercise and PCOS group with and without aerobic exercise (called NC, EC, PC, and PE group, respectively), and they were surprised to find that levels of TNF-α, IL-6, and Leptin were reduced in the PE group compared to the PC group. Thus, they speculated that aerobic exercise improved the inflammatory status of PCOS by reducing Leptin resistance. Aerobic exercise reduces internal inflammation by reducing Leptin resistance (82), and high-intensity interval training (HIT) reduces circulating Leptin levels in obese women and adolescents (both men and women) with risk factors for type 2 diabetes (83, 84). But things change when it comes to the obese PCOS (85). This may be due to the interaction between Leptin and PCOS, and the formation of PCOS will regulate the expression of Leptin, making it inconsistent with physiological conditions.

Similar to Adiponectin, contribution of ethnicity to the association of gene variants of Leptin receptor with PCOS had been clarified in a case control study (86), which showed that the rs1137100 was negatively associated with PCOS in Tunisians and Bahraini women, whereas rs2025804 was associated with PCOS only in Tunisians.

### Apelin

Boucher et al. demonstrated that Apelin secreted from human adipocytes, first extracted from bovine stomachs and so named in 1998 (87), is essentially an adipokine in 2005 (88). Located on the long arm of the human X chromosome, Apelin gene expresses a precursor peptide consisting of 77 amino acid residues, which is formed by proteolytic enzymes into different protein subtypes. The main biologically active forms are Apelin-36 and Apelin13 (89), with the Apelin-13 as the most common subtype in human plasma (90). Apelin is widely distributed in central and peripheral tissues, such as pituitary, heart, lung, kidney, breast, and adipose tissues, and can be transmitted by putative receptor protein related to AT1 in these tissues, regulating a wide range of physiological activities such as energy metabolism, body fluid balance, and regulation of food intake and angiogenesis (91).

Apelin regulates human gonads by initiating signaling pathways, such as mitogen-activated protein kinase3/1(MAPK3/1), protein kinase B(AKT), and AMPK *via* binding the Apelin receptors. Animal and cell line studies to date have revealed the role of Apelin in regulating the development of gonadal axis, ovarian angiogenesis, and follicles development (92–96). Apelin and its receptor are mainly expressed in granulosa cells, cumulus cells, and follicular membrane cells of the human ovary, but less in oocytes. In addition, the concentration of Apelin in plasma is lower than that in follicular fluid, and there is a reason to speculate that follicular Apelin is partly derived from granulosa cells to regulate granulosa cell function by paracrine and/or autocrine (97).

Bongrani et al. (98) showed that the protein and mRNA level of Apelin and its receptors in ovarian follicular fluid and granulosa cells of PCOS patients increased compared with the normal control group, with the elevated Apelin and its receptor being positively correlated with the number of preantral follicles. Insulin *in vivo* inhibits the production of IGF-1 binding protein and thus increases the biological activity of IGF-1. In PCOS patients, hyperinsulinemia caused by IR increases the activity of IGF-1 in the ovary, and IGF-1 promotes production of estrogen by stimulating Apelin. It was also reported that Apelin had a high expression in granulosa cells and follicular fluid of women with PCOS (99). Recombinant human Apelin-13 and Apelin-17 promote ovarian basal estrogen, progesterone, and insulin-like growth factor 1(IGF-1) secretion. The same study also showed that APLN increases IGF1-induced steroid production in human primary luteinized granulosa cells by increasing the hydroxysteroid dehydrogenase (HSD-3β) protein expression and activating the mapK3/1, and Akt pathways (99), implicating the effect of Apelin in the development of follicles. Vascular endothelial growth factor (VEGF) is critical for ovarian follicular angiogenesis and normal reproductive function. IGF-1 and VEGF directly or indirectly affect the ovary function of patients with PCOS in terms of cyst formation and angiogenesis by the Apelin system (100–102). Progesterone stimulated the expression of Apelin receptors in granulosa cells and hyperinsulinemia could stimulate Apelin and VEGF expression in PCOS. The expressions of insulin, Apelin, and VEGF (93, 103) were increased in this case (104), whereas Apelin receptors were significantly decreased because of the luteal phase defect (LPD) due to IR. Therefore, inefficient combination of APLN to the receptors is related to the pathogenesis of LPD in patients with PCOS. Two recent studies have shown that exercise affects Apelin levels *in vivo (* 105, 106). So, we do not deny that some studies have reached different conclusions, possibly due to the age assessment methods for genetic characteristics of exercise populations and the heterogeneity of PCOS itself.

### Resistin

Originally found in the white adipose tissue of mice, Resistin was so named because of its insulin-resistant effect, and was induced to produce and inhibit adipocyte differentiation during adipocyte differentiation (107). Located on the chromosome 19p13.3 with a span of 1369 bp, the Resistin gene consists of three introns and four exons (108), encoding a 12.5 kDa polypeptide with 108 aa mature fragments (109). Subsequent studies have shown that human macrophages, adipose tissue, oocytes, and granulosa cells at all developmental stages and follicular membrane cells of large follicles express Resistin at the mRNA and protein levels (110, 111). Thus far, four kinds of Resistin receptors have been identified: a subtype of Adenylate cyclase-associated protein 1 (CAP1), transmembrane Toll-like receptor 4 (TLR4), subtype of decorin (ΔDCN), and receptor tyrosine kinase-like orphan receptor 1 (ROR1), involved in the pathophysiological processes such as IR, inflammation, and apoptosis (112) through CAP1-cAMP-PKA/NF-kβ and TLR4-TIRAP/MyD88-JNK/p38 (113).

Although results of published studies are still inconclusive, the analysis by Raeisi et al. (114) revealed that, overall, the Resistin level was significantly higher in PCOS women compared with healthy controls, independent of obesity status. As a nonnegligible clinical manifestation of PCOS patients, HA may be related to the increased level of Resistin in serum (115). Further *in vitro* experiments showed that serum Resistin could directly promote formation of testosterone in follicular membrane cells of PCOS patients by enhancing the activity of 17α hydroxylase (116). Moreover, the follicular membrane cells of normal developing follicles also respond to Resistin stimulation. Therefore, the lack of correlation between Resistin and serum total testosterone in normal controls may be due to the existence of a substance inhibiting Resistin in normal ovaries.

Messini et al. (117) found that Resistin inhibited the estradiol and progesterone secretion of human corpus luteum granulosa cells stimulated by follicle stimulating hormone under normal physiological conditions. We hypothesized that a high level of Resistin in PCOS patients may enhance inhibition of estrogen progesterone secretion and thus cause the clinically observed low progesterone state in PCOS patients based on the results above. Nevertheless, the mechanism *via* which Resistin can affect the production of estradiol and/or progesterone in the granulosa cells is not clear. Combination of Resistin and decorin induced cell proliferation and migration, resulting in the expansion of white adipose tissue (118). Increased white adipose tissue increases the secretion of various adipokines, such as Chemerin, which may influence the occurrence and development of PCOS from another direction. It has become appreciated that PCOS encompasses chronic inflammatory state all over the body, including the ovaries, and Toll-like receptor-4 has also been shown to be expressed in bovine ovarian granulosa cells (119). Moreover, it was found that human macrophages generated Resistin and directly participated in the regulation of inflammation. When the level of serum Resistin increased, macrophages were activated to produce TNF-α, IL-6, IL-12, and other pro-inflammatory factors (120, 121). Regrettably, there have been very few studies on the expression of TLR4 in ovarian granulosa cells as well as the level of Resistin and local inflammation of ovary in PCOS patients. Therefore, further well-designed studies with large sample sizes should be performed to examine the circulating levels of Resistin and its role in PCOS.

### Visfatin

First cloned from human peripheral blood lymphocyte cDNA library in 1994 as a cytokine, Visfatin was also named pre-B-cell colony enhancer factor (PBEF) (122). As a 52-kDa secreted protein, Visfatin is expressed mainly in human visceral fat, as well as bone marrow, liver, and muscle (123). In mammals, Visfatin exists in two forms, intracellular and extracellular, namely iNAMPT and eNAMPT (124), identified as an important enzyme (nicotinamide phosphoribosyl transferase) involved in metabolism (125, 126).

Studies showed that granulosa cells, cumulus cells, oocytes, and follicular membrane cells of human ovary can secret Visfatin (127). It has also been proved that the number of oocytes retrieved was increased with increasing levels of Visfatin in follicular fluid (128), further substantiating a positive impact of Visfatin on female reproduction. There was no association in Visfatin levels between plasma and follicular fluid, and circulating Visfatin concentration seems to have little effect on Visfatin concentration in follicular fluid (128), suggesting little communication between blood and follicular fluid Visfatin and an important role of the local Visfatin in ovary. Visfatin is involved in local ovarian energy metabolism as a key enzyme in nicotinamide adenine dinucleotide (NAD) biosynthesis and may thus affect follicular development. In addition, recombinant human Visfatin promotes human granulosa cell proliferation and IGF-1-dependent steroid hormone production (127). In bovines, Visfatin is involved in GC steroidogenesis, proliferation, and oocyte maturation, as well as increasing the secretion of E2 secretion accompanied by an increase in StAR and HSD-3β expression (129).

There are contradictory reports on the levels and expression of Visfatin in PCOS subjects. More studies tend to favor a higher level of ovarian Visfatin in PCOS (130–132). In addition to adipocytes, inflammatory cells, such as macrophages, have also been shown to secrete Visfatin, which may suggest PCOS as a chronic low-grade inflammatory condition (133). Its elucidated insulin-like effects were consistent with the high levels observed in PCOS (134). A recent study found that FK866, an inhibitor of Visfatin, improved letrozole-induced pathological state of hyper-androgen PCOS mice, inhibiting androstendione and testosterone secretion, inhibiting ovarian cyst formation and apoptosis, but promoting luteal formation, and increasing local ovarian glucose content (131). In summary, whether Visfatin plays a role in the pathogenesis of PCOS is still under discussion and needs more reliable studies.

### Vaspin

Visceral adipose tissue-derived serpin (Vaspin) is a member of the serine protease inhibitor family, encoded by the SERPINA12 gene on the long arm of human chromosome 14 (14q32.1), and it is constituted by 395 amino acids as a 47-kDa protein secreted mainly by visceral and subcutaneous adipose tissue (135). Vaspin is considered to be closely related to lipid metabolism and IR (136) through binding to a 78-kDa glucose-regulated protein (GRP78) on the cell surface (137).

Several studies have shown that the circular Vaspin level is elevated in PCOS patients and plays a role in the process of IR among PCOS patients (138–140). In 2008, the first study about Vaspin in PCOS women was published (141), showing that circulating Vaspin and the transcript as well as translation levels of Vaspin in omental adipose tissue are significantly elevated in women with PCOS. In the same study, the *in vitro* experiments demonstrated that glucose stimulates the secretion of Vaspin in omental adipose tissue. Also, the serum Vaspin levels in PCOS women decreased significantly after 6 months of metformin treatment. In a study of serum Vaspin levels in different types of PCOS (142), researchers found that serum Vaspin level has an independent correlation with BMI and HOMA-IR. Moreover, they observed the phenomenon that PCOS patients diagnosed with all three criteria (also named the “classical” phenotype) have the highest serum Vaspin levels, indicting the correlation between PCOS severity and Vaspin. It was shown that the Vaspin rs2236242 variant in the Iranian population was associated with metabolism in obese PCOS patients, and the A allele reduced the risk of PCOS compared to the T allele, even though the association disappeared after adjusting for BMI (143). Bongrani et al. (144)detected Vaspin for the first time in the human ovary to improve the granulosa cells function concentration-dependently, which gives us evidence that Vaspin may have a regulatory role on granulosa cells in PCOS. In addition, a prospective case control study showed that those infertile PCOS patients who had successfully induced ovulation after clomiphene treatment had significantly lower serum Vaspin levels, suggesting that serum Vaspin level may be a useful marker for prediction of ovulation induction (140). Taken together, we can conclude that elevated serum Vaspin levels in patients with PCOS are associated with their metabolism, mainly IR and BMI, and may act as a positive factor in regulating ovarian granulosa cell function, whereas the effect of Vaspin on the outcome of assisted conception in infertile patients with PCOS remains to be further investigated.

### Lipocalin 2

Lipocalin 2 is a novel adipokine (145), a member of the Lipocalin superfamily, whose coding gene is located on human chromosome 9 with the functional protein consisting of 198 amino acids. The major source of Lipocalin 2 expression is white adipose tissue (WAT) (146). Megalin, a glycoprotein located on the surface of the cell membrane, has been proposed for human Lipocalin 2, and they bind to each other to exert biological effects (147). Lipocalin 2 has been illustrated to play a role in the pathophysiological processes such as cell differentiation, apoptosis, organogenesis, and inflammation (148). In addition, it has been shown to be significantly upregulated in obesity and T2DM (149).

No meta-analysis of Lipocalin 2 levels in serum of PCOS patients is available thus far, and there are inconsistent results in the published studies. Two of them showed an elevated serum Lipocalin 2 level in PCOS patients (150, 151), correlated with serum insulin levels, HOMA-IR, and free testosterone. Gencer et al. found that serum Lipocalin 2 levels were lower in PCOS compared to those with normal ovulation (152). Patients with PCOS are known to be at an increased cardiac risk, but no correlation has been shown between Lipocalin 2 levels and early detection of atherosclerosis in PCOS patients. Three other studies showed no significant difference between patients with PCOS and the control group (153–155), which illustrated that Lipocalin 2 levels were higher in overweight/obese PCOS patients than in normal weight PCOS and control patients. Lipocalin 2 was independently associated with BMI, and weight loss led to a decrease in Lipocalin 2 levels. In addition, the study by Martínez-García et al. added conviction to the masculinization of adipokine secretion in PCOS patients (156) when comparing the expression of Lipocalin 2 of subcutaneous (SAT) and visceral adipose tissue (VAT) between severely obese men and hyperandrogenic women presenting with PCOS submitting to bariatric surgery. They found that in men and women with PCOS, Lipocalin 2 expression was higher in VAT than in SAT; in the control group, the contrary is also the case. Two other studies yielded lower Lipocalin 2 levels in PCOS patients compared to controls (157, 158). Because Lipocalin 2 activates atherosclerotic plaques, low Lipocalin 2 levels are thought to be protective in patients with PCOS (158). Although attenuated after adjustment for waist and fasting glucose, Lipocalin 2 remained positively associated with diabetes progression in the PCOS group (157). Thus far, there are no studies on the association of Lipocalin 2 gene polymorphisms with PCOS. Despite different results, we can see that Lipocalin 2 may be present as a protective factor in PCOS. However, works on expounding the mechanisms between Lipocalin 2 and PCOS remain insufficient until now.

### Omentin

Omentin, a novel adipokine, was originally identified from the human omental adipose tissue cDNA library and thus named (159). The human Omentin gene is located on chromosome 1q21.3 and encodes a 313 amino acid protein. Of note, visceral adipose tissue rather than subcutaneous adipose tissue often expresses Omentin-1 preferentially and abundantly (160). Omentin is a secretory adipokine with pleiotropic effects, and Omentin plays a positive regulatory role in IR, inflammation, and regulation of endothelial function and so on (161, 162).

A meta-analysis in 2017 focusing on circulating Omentin levels in women with PCOS showed significant low levels of Omentin in patients with PCOS (163), and numerous studies have demonstrated the role of Omentin in the pathological processes of inflammatory state, IR, and steroid hormone production in PCOS (162, 164, 165). Cloix et al. identified the expression of Omentin in human ovarian granulosa cells for the first time in 2014 (165). They found similar levels of Omentin in plasma and follicular fluid in control patients but significantly higher levels of Omentin in follicular fluid than in plasma in patients with PCOS. They also demonstrated the function of human granulosa-lutein cells to secrete Omentin under insulin stimulation. In addition, the treatment of human recombinant Omentin increased nicotinamide phosphoribosyl transferase (NAMPT), IGF-1-induced progesterone, and estradiol secretion, and this effect disappeared when NAMPT was knocked down. Another article aimed at studying the regulation of Omentin secretion *in vivo* and *in vitro* revealed low expression of Omentin in omental adipose tissue of women with PCOS and demonstrated that insulin can reduce Omentin expression in a dose-dependent manner (166). Another case-control study showed that after adjusting for BMI, Omentin levels in PCOS individuals with IR were lower than in those without IR. Furthermore, Omentin was negatively correlated with BMI, HOMA-IR, and fasting insulin (164). Taken together, we can speculate that insulin regulates Omentin in different ways at different sites which explains, to some extent, the opposite direction of changes in Omentin in serum and follicular fluid of PCOS patients. A cross-sectional study (162) conducted by Franik et al. revealed that plasma Omentin levels were reduced and associated with the high circulating TNF-α and low IL-6 levels in patients with PCOS. We know that IL6 has both pro-inflammatory and anti-inflammatory properties. While it is not clear whether Omentin has a driving or inhibitory effect in the inflammatory state of PCOS, it does affect its inflammatory state based on the data provided by Franik et al. It was shown that serum Omentin levels were negatively correlated with free testosterone levels and tended to decrease as free testosterone levels increased (167), suggesting that the increase in free testosterone in PCOS patients led to a decrease in the level of the protective factor-Omentin. PCOS patients with HA may be accompanied by hirsutism. In a study on the relationship between female hirsutism and serum Omentin (168) measuring the serum Omentin and free testosterone in 30 patients with hirsute PCOS, 30 patients with idiopathic hirsutism and 25 healthy control women, a significant inverse correlation was revealed in the serum testosterone level with Omentin, and the idiopathic hirsutism group had the highest significant level of serum Omentin level. This may indicate that Omentin may not play a role in the development of hirsutism in PCOS patients, but is directly related to hyperandrogenism. Additionally, treatment with metformin and oral contraceptives significantly increased serum Omentin levels in PCOS patients (169, 170). Tan et al. and colleagues (170) also found that serum VEGF levels were elevated in PCOS patients and decreased with the reduction of Omentin levels after metformin treatment. VEGF, an essential factor for angiogenesis, plays an important role in ovarian polycystic-like changes, and this study verified that Omentin may play a role in inhibiting polycystic ovary morphology (PCOM) changes. This may give us enlightenment on improving assisted conception outcomes in patients with PCOS.

## Summary and Prospects

**Figure 1:**
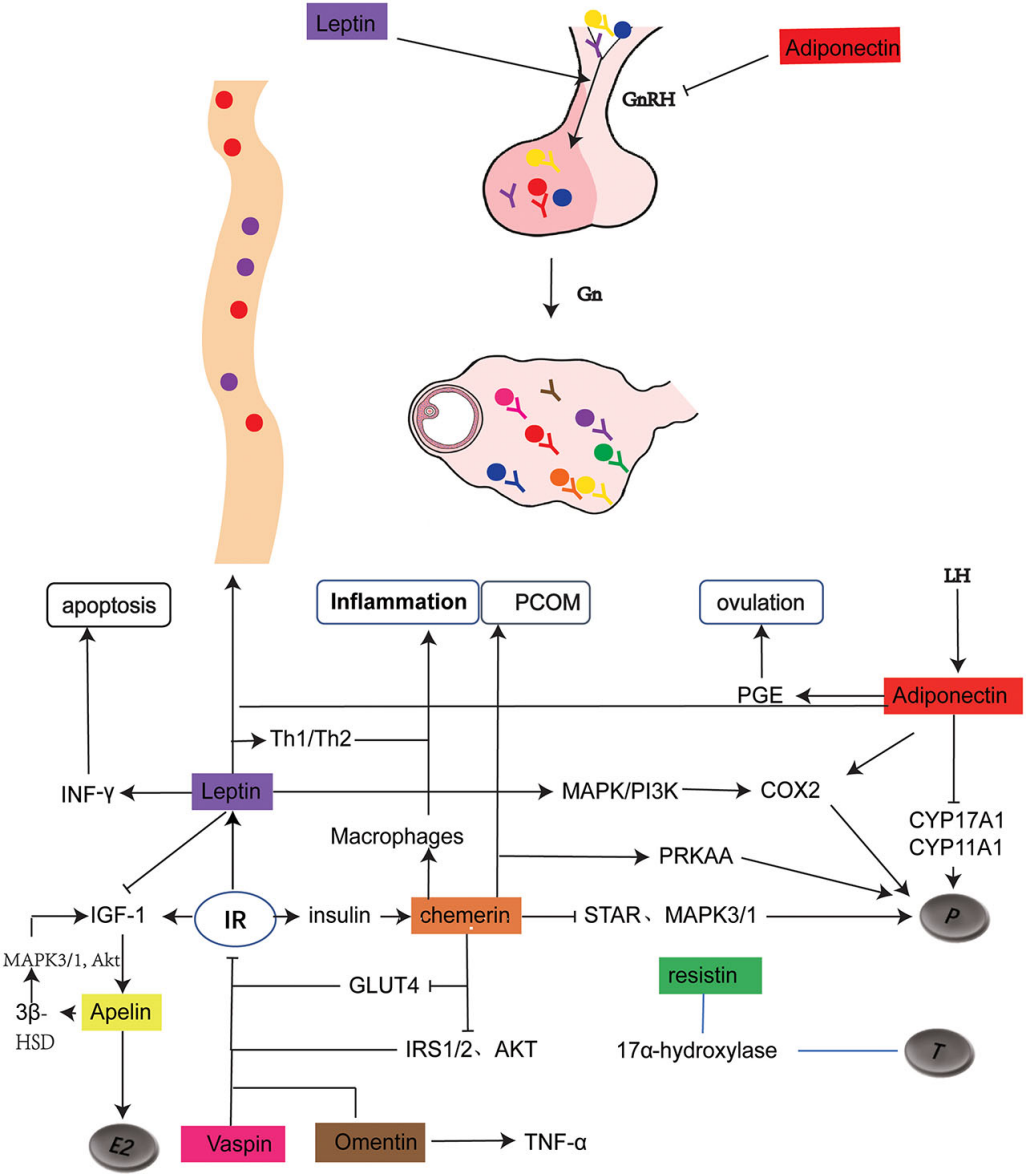
Schematic representation of adipokine expression and its role in PCOS. The solid dots of different colors represents the distribution of adipokines, with the corresponding Y as their receptors .
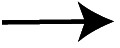
 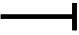 represents for promotion and inhibition respectively. As shown in the figure, leptin and adiponectin regulates hypothalamic secretion of GnRH. Leptin, together with Adiponectin and Chemerin regulates progesterone production. Resistin is related to testosterone production without clear mechanism. Chemerin and Omentin are associated with inflammation. Chemerin, Apelin, Leptin, Vaspin, Omentin play a role in regulating IR. In addition, Adiponectin is closely related to ovulation and Chemerin is involved in the formation of PCOM.GnRH, gonadotropin-releasing hormone; Gn, gonadotropin; LH, luteinizing hormone; P, proges-terone; T, testosterone; E2, Estrodiol; PCOM, polycystic ovary morphology; PGE, prostaglandin E; MAPK, mitogen-activated protein kinase; COX2, cyclooxygenase-2; CYP17A1, cytochrome P45017A1; CYPI1A1, cytochrome P45011A1; IGF-1, insulin-like growth factor 1; IR, insulin resistance; IRS, Insulin receptor substrate; AKT, protein kinase B; INF- y , Interferon- y ; 3 8 -HSD, 3 6 -hydroxysteroid dehydrogenase; GLUT4, glucose transporter4.

The occurrence of PCOS involves multiple pathological processes and is influenced by more than one factor just like its various clinical manifestations. Just as there are no isolated individuals in the world, all the adipokines mentioned above may interweave and influence the development of PCOS (**Figure 1**). It is noteworthy that Adiponectin, Leptin, and Apelin are all beneficial under normal physiological conditions, possibly with different trends in the body fluids of PCOS patients. Adipokines exhibit compensatory overexpression due to the decline in their receptors, such as Leptin and Apelin. Although each adipokine affects more or less all aspects of PCOS, the focus is different. Chemerin worsens the IR of PCOS by affecting glucose utilization of granulosa cells. Adiponectin plays an important role in promoting ovulation and may be involved in maintaining negative feedback in the reproductive axis, but unfortunately, it may be declined in PCOS with unknown reason. Apelin maintains progesterone secretion in the luteal phase, but is limited by the reduction of its receptor caused by IR, the most common manifestation of PCOS. As for Resistin, Visfatin, and Metrnl, current studies are limited only to animals or cells, of which results are confined to the correlation of clinical or laboratory data. When it comes to Vaspin, Lipocalin 2, and Omentin, most of their current studies in PCOS patients are related to IR, even with other different priorities. Omentin plays a role in the regulation of inflammatory status and steroid hormone production in patients with PCOS, whereas Vaspin has been shown to occupy an important position in the proliferation, viability, and function of ovary granulosa cells. Due to the dynamic changes in the body and the complex interplay between hormones, it is not easy to understand all the specific mechanisms involved, and hence, the underlying mechanism needs to be confirmed further.

PCOS, as a highly heterogeneous syndrome, is still controversial in its diagnosis, and its pathogenesis is not yet clear, making it a hot topic of continuous research for so many years. Adipokines, on account of their significant roles in energy metabolism, inflammation, insulin-resistance, cell senescence, and apoptosis, together or independently affect the pathological process of PCOS (**Table 1**), and have become one of the most fascinating areas of PCOS exploration. Current studies have confirmed that a variety of adipokines are differentially expressed in PCOS and non-PCOS populations, and are related to ovarian angiogenesis, steroid hormone-generation, follicular development, and granulosa cell apoptosis. Notwithstanding, most studies are limited to defining the superficial correlation rather than clarifying the underlying mechanism, for which we still have a long way to go.

**Table 1:** Adipokines and their main effects in PCOS.

Adipokines	Site of production	Circulating levels	Effects in PCOS
Adiponectin	White adipose tissue	↓	Regulates gonadal axis hormone secretion, promotes ovulation, and improves insulin resistance
	Pituitary gland		
	Ovary		
Chemerin	White adipose tissue	↑	Aggravates insulin resistance and reduces estrogen and progesterone expression
	Ovary		
Metrnl	White adipose tissue	↓	Negatively correlated with fasting glucose, fasting insulin, and HOMA-IR
Leptin	White adipose tissue	↑	Aggravates IR, promotes inflammation, induces granulocyte apoptosis, and regulates steroid hormone secretion
Apelin	White adipose tissue	→	Promotion of IFG-1-induced estrogen and progesterone secretion
	Hypothalamus		
	Pituitary gland		
	Ovary		
Resistin	White adipose tissue	↑	Inhibits estrogen and progesterone secretion, promotes testosterone production; activates macrophages to produce pro-inflammatory factors such as TNF-α
	Ovary		
Visfatin	Visceral adipose tissue	↑	Participates in local energy metabolism in the ovary, regulates steroid hormone production such as suppressing androgen production and promoting IFG-1-induced estrogen and progesterone secretion
	Hypothalamus		
	Pituitary gland		
	Ovary		
Vaspin	Visceral and subcutaneous adipose tissue, ovary	↑	Improves insulin sensitivity and regulates granulocyte function
Lipocalin 2	Visceral and subcutaneous adipose tissue	conflict	Associated with diabetes progression
Omentin	Visceral adipose tissue	↓	Regulates inflammatory status and reduces insulin resistance

↑, Increased circulating levels, ↓, Decreased circulating levels, →,equally circulating levels. are explained in the text. In the table, we only list the main sites of production and effects in PCOS.

## Author Contributions

PC and ZZ contributed to conceived and designed the review. PC wrote the paper. RJ and YL did the document retrieval. PC and MC draw the picture. LZ polished the paper. ZZ cheched the paper. All authors listed have made a substantial and intellectual contribution to the review and approved it for publication.

## Funding

This study was supported by Natural Science Foundation of Hebei Province (Beijing-Tianjin-Hebei Cooperation Special Project)(H2019206712).

## Conflict of Interest

The authors declare that the research was conducted in the absence of any commercial or financial relationships that could be construed as a potential conflict of interest.

## Publisher’s Note

All claims expressed in this article are solely those of the authors and do not necessarily represent those of their affiliated organizations, or those of the publisher, the editors and the reviewers. Any product that may be evaluated in this article, or claim that may be made by its manufacturer, is not guaranteed or endorsed by the publisher.
